# Structural characterization and conformational dynamics of alpha-1 antitrypsin pathogenic variants causing alpha-1-antitrypsin deficiency

**DOI:** 10.3389/fmolb.2022.1051511

**Published:** 2022-11-24

**Authors:** Noor Ahmad Shaik, Najla Bint Saud Al-Saud, Thamer Abdulhamid Aljuhani, Kaiser Jamil, Huda Alnuman, Deema Aljeaid, Nasreen Sultana, Ashraf AbdulRahman El-Harouni, Zuhier Ahmed Awan, Ramu Elango, Babajan Banaganapalli

**Affiliations:** ^1^ Department of Genetic Medicine, Faculty of Medicine, King Abdulaziz University, Jeddah, Saudi Arabia; ^2^ Princess Al-Jawhara Al-Brahim Center of Excellence in Research of Hereditary Disorders, King Abdulaziz University, Jeddah, Saudi Arabia; ^3^ Department of Biological Sciences, Faculty of Science, King Abdulaziz University, Jeddah, Saudi Arabia; ^4^ Department of Genetics, Bhagwan Mahavir Medical Research Centre, Hyderabad, India; ^5^ Department of Biotechnology, Acharya Nagarjuna University, Guntur, India; ^6^ Department of Clinical Biochemistry, Faculty of Medicine, King Abdulaziz University, Jeddah, Saudi Arabia; ^7^ Department of Genetics, Al Borg Medical Laboratories, Jeddah, Saudi Arabia

**Keywords:** SERPINA1 gene, alpha-1-antitrypsin, serpinopathies, molecular dyanmics, AATD

## Abstract

**Background:** Alpha-1 antitrypsin deficiency (A1ATD) is a progressive lung disease caused by inherited pathogenic variants in the *SERPINA1* gene. However, their actual role in maintenance of structural and functional characteristics of the corresponding α-1 anti-trypsin (A1AT) protein is not well characterized.

**Methods:** The A1ATD causative *SERPINA1* missense variants were initially collected from variant databases, and they were filtered based on their pathogenicity potential. Then, the tertiary protein models were constructed and the impact of individual variants on secondary structure, stability, protein-protein interactions, and molecular dynamic (MD) features of the A1AT protein was studied using diverse computational methods.

**Results:** We identified that A1ATD linked *SERPINA1* missense variants like F76S, S77F, L278P, E288V, G216C, and H358R are highly deleterious as per the consensual prediction scores of SIFT, PolyPhen, FATHMM, M-CAP and REVEL computational methods. All these variants were predicted to alter free energy dynamics and destabilize the A1AT protein. These variants were seen to cause minor structural drifts at residue level (RMSD = <2Å) of the protein. Interestingly, S77F and L278P variants subtly alter the size of secondary structural elements like beta pleated sheets and loops. The residue level fluctuations at 100 ns simulation confirm the highly damaging structural consequences of all the six missense variants on the conformation dynamics of the A1AT protein. Moreover, these variants were also predicted to cause functional deformities by negatively impacting the binding energy of A1AT protein with NE ligand molecule.

**Conclusion:** This study adds a new computational biology dimension to interpret the genotype-protein phenotype relationship between S*ERPINA1* pathogenic variants with its structural plasticity and functional behavior with NE ligand molecule contributing to the Alpha-1-antitrypsin deficiency. Our results support that A1ATD complications correlates with the conformational flexibility and its propensity of A1AT protein polymerization when misfolded.

## Introduction

A1ATD is a rare autosomal recessive disease in which low levels of circulating alpha-1 antitrypsin enzyme in the plasma promote degenerative or destructive changes in the lung (Ferrarotti et al., 2018). A1AT protein is a serine protease inhibitor produced in the liver. This enzyme binds to different enzymes, including neutrophil elastase, that cleaves them and gets cleaved by them in a suicidal fashion (Vissers et al., 1988). A proportion of A1ATD patients develop liver cirrhosis, which may be caused by aggregates of alpha-1-antitrypsin proteins (Köhnlein and Welte, 2008). While it is often undiagnosed (Quinn et al., 2020), it causes emphysema, which can be exacerbated by tobacco smoke (Tejwani and Stoller, 2021). It affects 1 in 2,500 people of European ancestry (Greulich et al., 2017).

The A1ATD is caused by different pathogenic variants in the SERPINA1 gene, which is located on the long arm (q) of chromosome 14 at 32.1 and consists of 7 exons. It encodes a 394 amino acid long polypeptide, which acts as a molecular mouse trap that binds and blocks the function of a variety of proteases. The amino acid substitutions in A1AT may also alter the cellular function by forming polymer aggregates of the protein, causing liver and lung damage (Duvoix et al., 2014). Many pathogenic alleles in SERPINA1 gene, also designated as protease inhibitor (PI), have already been described in A1ATD patients (Foil, 2021). The misfolding and aggregation of this serpin family member is thermodynamically favorable since the protein’s native conformation is transient and built to be cleaved to reach stability (Cho et al., 2005).

The effects of missense variants on A1AT’s structure and function are not yet well resolved. Classical *in-vivo* and *in-vitro* approaches to study the molecular characterization of pathogenic variants are time and cost-consuming. Alternative “*in silico*” approaches, owing to their sensitivity, specificity, and accuracy, act as pre-screens for laboratory studies (Shaik et al., 2020a; Shaik et al., 2020b; Awan et al., 2021). In this regard, a growing number of computational methods can effectively predict variant pathogenicity and stability, visualize their structures, map the conserved domains, compare their secondary structures with the wildtype protein, and simulate their ability to bind with a substrate. Therefore, it is aimed to utilize these computational biology methods to study clinical missense variants to expand the knowledge on the nature of structural defects and conformational dynamics affecting the A1AT’s function.

## Materials and methods

### *SERPINA1* variant data collection and curation

The molecular details of the *SERPINA1* gene, including the nucleotide sequence, chromosome position, transcript, and the corresponding amino acid sequence, were obtained from the National Center for Bioinformatics (NCBI) (www.ncbi.nlm.nih.gov) and Ensembl databases (www.ensembl.org). The A1ATD causative variants were collected from the DisGenNET platform (https://www.disgenet.org), which is a webserver that contains disease-associated variants gathered from scientific literature, genome-wide association studies (GWAS) catalogues, and animal models (Piñero et al., 2021). The reported variants are downloaded in a list in Microsoft Excel format. Then, only the missense variants associated with the A1ATD phenotype were selected for further analysis, after sorting and filtering by Microsoft Excel 2019. Table 1 shows the molecular details of the selected *SERPINA1* missense variants.

**TABLE 1:** Molecular details of alpha-1-antitrypsin causative SERPINA1 missense variants.

#	dbSNP ID	Clinvar ID	Chromosomal position	c.DNA position	Exon	Amino acid change	Codon change	MAF
1	rs1555369172	VCV000444040.1	14:94383011-94383011	c.274T>C	2/5	F76S	tTc/tCc	—
2	rs55819880	VCV000017992.2	14:94383008-94383008	c.277C>T	2/5	S77F	tCc/tTc	—
3	rs756773408	VCV000444044.1	14:94382592-94382592	c.693G>T	2/5	G216C	Ggc/Tgc	—
4	rs1566753480	VCV000626306.1	14:94380955-94380955	c.880T>C	3/5	L278P	cTg/cCg	—
5	rs17580	VCV000626305.1	14:94380925-94380925	c.910A>T	3/5	E288V	gAa/gTa	0.0196
6	rs1555367891	RCV000512630.1	14:94378633-94378633	c.1120A>G	5/5	H358R	cAt/cGt	—

### Variant pathogenicity predictions and conservation analysis

The selected missense variants were uploaded into the Ensembl (www.ensembl.org) variant pathogenicity predictor (VEP) to assess their pathogenic potential. This webserver allows using multiple tools that measure whether a variant can be considered deleterious or not based on different features like whether it is located in a evolutionarily conserved sequence across species, or whether it causes structural and stability differences, *etc.* (Adzhubei et al., 2010). The data can be entered as a variant ID, VCF file, or nomenclature notation of HGVS coordinates. The output can be in text or html format. In this study, six tools, including Combined Annotation Dependent Depletion (CADD), Scale-invariant Feature Transform (SIFT) (Ng and Henikoff, 2003), Polymorphism Phenotyping (PolyPhen) (Adzhubei et al., 2013), Mendelian Clinically Applicable Pathogenicity (M-CAP) (Jagadeesh et al., 2016), and Functional Analysis through Hidden Markov Models (FATHMM) (Rogers et al., 2018), REVEL (rare exome variant ensemble learner) were used to evaluate the pathogenicity of variants (Ioannidis et al., 2016). SIFT predicts pathogenicity based on alteration in conserved regions of the nucleotide sequence (Shaik et al., 2020b; Shaik et al., 2021; Alharthi et al., 2022; Bima et al., 2022). PolyPhen predicts the variant effects based on the nucleotide sequence and changes in protein structure. CADD predicts the effects based on the integration of several parameters, including sequence context, evolutionary constraints on the genome, and epigenetic calculations. The M-CAP combines the predictions of PolyPhen, SIFT, and CADD together. FATHMM predicts the consequences based on combining sequence conservation within Hidden Markov models (HMMs) to depict the alignment of homologous sequences and conserved protein domains. REVEL is an ensemble technique for estimating the pathogenicity of missense variants utilizing the following methods: MutPred, FATHMM, VEST, PolyPhen, SIFT, PROVEAN, MutationAssessor, MutationTaster, LRT, GERP, SiPhy, phyloP, and phastCons. REVEL was trained exclusively on rare pathogenic and neutral missense variants (Ioannidis et al., 2016). To examine the amino acid sequence conservation pattern of A1AT gene across related species (8 primates), we have performed multiple sequence alignment using Clustal Omegat (https://www.ebi.ac.uk/Tools/msa/clustalo/).

### 3D structure mapping and superimposition

The PDB database contains the full-length x-ray crystallographic structure of the natural human native A1AT protein at 1.8 Å (PDB ID: 3NE4 chain A). This structure was used as a template to construct A1AT protein variants using Modeller-homology model software. The full-length amino acid sequence of A1AT retrieved from the NCBI database (accession number CAJ15161.1) was used to provide input to the Modeller tool to construct tertiary structural models of A1AT variants. The Modeller is a readily available online tool that relies on protein NMR to meet spatial restrictions, using sets of geometrical requirements to establish atomic positions in protein models by generating probability density functions. This approach matches the input sequence with target amino acid sequences and the structure of the template protein. Using the steepest descent force field method in the GROMACS software, energy of three-dimensional mutant A1AT structure was optimized with steepest descent energy minimization method. All 3D models, including the wildtype and mutated structures, were viewed, and analyzed through Pymol2 software.

### Structural deviation and secondary structure analysis of A1AT variants

The structural deviation of mutated proteins is a reliable metric to determine how an amino acid change affects the overall structure of the protein. The structural deviation of proteins is measured in form of RMSD values, which were computed with the Pymol2 software. The smaller the RMSD value, higher similarity in both structures is predicted. These RMSD values were estimated by superimposing each mutated model with the corresponding wildtype structure. To perform the secondary structure analysis, each mutated amino acid sequence was created manually in text format *via* manual amino acid substitution. Then, along with the wildtype, all mutated amino acid sequences were entered into the Netsurfp 2.0 web tool (https://services.healthtech.dtu.dk/service.php?NetSurfP-2.0) to generate secondary structure representations. These secondary structures were then analyzed to see if variant induced changes occurred at the secondary structural element like α-helix, β-pleated sheet or loop.

### Stability analysis of A1AT variants

The missense variants were analyzed for their ability to increase or decrease the stability of the protein by using the DUET server (http://biosig.unimelb.edu.au/duet), which predicts stability scores in ΔΔG in kcal/mol. This server uses variant cutoff scanning matrix (mCSM) and site-directed mutator (SDM) methods for estimation of a DUET score, which is calculated based on integrated scores of both the aforementioned methods (Pires et al., 2014). The input to this server can be the four-letter code of the wildtype protein, or the PDB structure, together with the amino acid change and the affected chain letter code. If the value is positive, the variant is making the structure more stable, and if it is negative, it is making the structure less stable.

### Molecular dynamics simulation

The MD simulation of the wild and mutant protein models was done using the Desmond MD tool (Bowers et al., 2006; Release, 2017). The simulation systems were first prepared by applying the SPC/E solvation model to an orthorhombic box with a boundary distance of 10 Å. The system for all the models was neutralized by adding 10 Na^+^ and 0.15 M Na^+^, Cl^−^ ions using the OPLS3e forcefield. Energy minimization of the prepared solvent systems was minimized using the steepest decent method at 5,000 steps. Further, the minimized systems were equilibrated in constant temperature and volume (canonical or NVT) and constant temperature and pressure (NPT) ensembles using a “relax model system” before the simulation. In the initial steps, the energy minimized systems are simulated in the NVT ensemble with Brownian dynamics at 10K temperature for 100 ps and 12 ps with restraint on solute heavy atoms. In NPT ensemble systems, no restraints were on heavy atoms at 10 K and 300 K temperatures for 12 ps and 24 ps, respectively. The fully equilibrated systems were finally subjected to 100 ns unrestrained MD simulations in an NPT ensemble with 1.01325 pKa (pressure) and 300 K temperature. The 25 ns trajectories were recorded during the simulation period for post MD analysis.

### A1AT—NE protein computational binding assay

The binding affinity between A1AT (both native and mutant forms) with *NE* (neutrophil elastase) proteins was analyzed using the ClusPro molecular docking online tool (https://cluspro.org). The input for this tool is the protein and ligand files in PDB format. The other options were set as “default.” This tool utilizes the PIPER algorithm where the center of mass of the receptor remains fixed, and the ligand molecule is rotated in a variety of positions to determine the best fit (Kozakov et al., 2013; Kozakov et al., 2017; Vajda et al., 2017; Desta et al., 2020). The resulting models were compared and only the best scores, depicted as 0 for each model, were selected for downloading and visual simulation in Pymol2. Moreover, the lowest negative energy score outputs were recorded along with center scores for the analysis of binding energy variation.

## Results

### Predicting missense deleterious variants and evolutionary conservation analysis

The deleterious effects of all 6 (F76S, S77F, L278P, E288V, G216C, and H358R) missense variants were measured with various “*in silico*” prediction tools; SIFT, PolyPhen, CADD, FATHMM M-CAP, and REVEL in the Variant Effect Predictor (VEP) web server from Ensembl. One of the variants (L278P) was predicted to be pathogenic by all tools except CADD with a C-score of 29.1, which was close to the cutoff value of 30 (Table 2). The pattern of amino acid sequence conservation implies that all A1AT variants were mapped to the evolutionarily conserved region (F76S, S77F, L278P, E288V, G216C, and H358R) (Supplementary Figure S1). A1AT in humans has a relatively close phylogenetic link with bonobos and chimpanzees, but it is distinct from mouse lemurs, based on the phylogenetic relationships among 12 species of primates that are closely related to one another.

**TABLE 2:** The pathogenicity prediction output of different computational tools of the SERPINA1 missense variants.

#	Amino acid variant	SIFT	PolyPhen	CADD	FATHMM	M-CAP	REVEL
1	F76S	0.00	1.000	32.0	0.86815	0.75948	0.98304
2	S77F	0.00	1.000	31.0	0.95561	0.82026	0.99571
3	G216C	0.00	1.000	35.0	0.91589	0.83675	0.9706
4	L278P	0.00	0.999	29.1	0.90962	0.88091	0.96096
5	E288V	0.00	0.996	32.0	0.89496	—	0.8882
6	H358R	0.00	0.990	26.8	0.92158	0.80576	0.94428

SIFT: < 0.01 = damaging, PolyPhen: > 0.5 = damaging, CADD: > 30 damaging, FATHMM >0.5 = damaging, and M-CAP > 0.5 = damaging; REVEL >0.5 = damaging.

### 3D modeling and stereochemical quality assessment of wildtype protein

**FIGURE 1:**
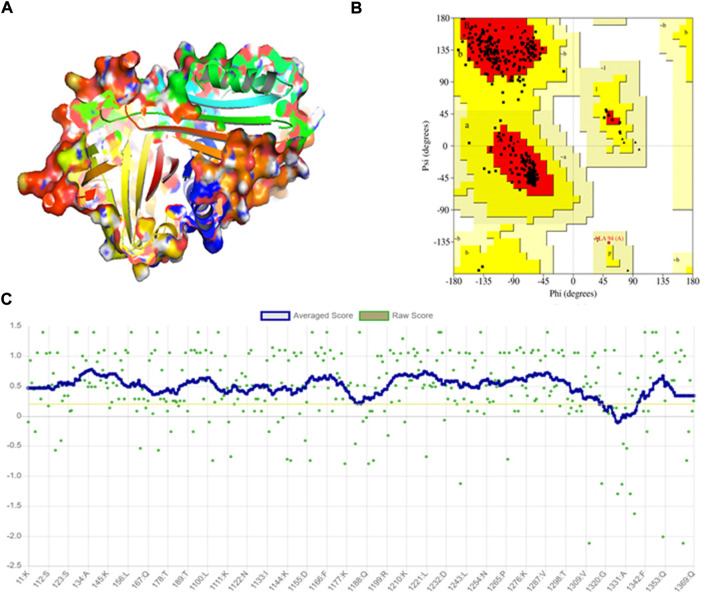
Modeling and stereochemical quality analysis of A1AT wildtype protein. **(A)** Energy-minimized wildtype model of A1AT protein generated by Pymol2. **(B)** Ramachandran Plot for the A1AT energy-minimized wildtype structure representing amino acid deposits in the center and non-center areas of the protein (90.9%–8.8%, respectively). **(C)** Hydrogen bonding estimation of energy-minimized A1AT wildtype protein, indicating a good quality structure.

The crystal structure of A1AT is in metastable native fold form and consists of three sheets, nine helices, and a reactive center loop held at the apex of the protein. The amino acids 357 to 359 allowed the RCL region extend β-stand conformation (stressed external loop) and stabilize the structure by forming slat bridges between P5 Glu and Arg 196, 223, and 281. Upon 3D modeling of the wildtype protein in Pymol2, the resultant structure was subjected to energy minimization to remove bad physical configurations. This was achieved using the Modeller tool. The energy minimization output was shown to be −17874.756 kJ/mol, which shows the successful removal of unwanted bonding patterns from the 3D model (Figure 1A). SAVES v.6.0 was used in the analysis of protein structure stereochemical quality assessment through a Ramachandran plot (Figure 1B), which revealed that a small number of amino acids have their φ (phi), ψ (psi) angles in the non-core areas of the protein. The percentage of amino acid deposits in the center and non-center areas of the protein is 90.9%–8.8%, respectively. The hydrogen bond estimation (DSSP) algorithm also revealed the output for hydrogen bonding as a graphical representation (Figure 1C), which demonstrates the good quality of the built protein model. The average DSSP score falls between 0 and 1, indicating a good quality protein structure.

### Structural deviation analysis of A1AT variants

The stereochemical analysis of the energy minimized A1AT mutant protein models (F76S, S77F, L278P, E288V, G216C, and H358R) showed that approximately 99.7% of the amino acids fall in the allowed region and only 0.03% in the disallowed region (Figure 1B). Moreover, it displayed good overall structural quality through the Procheck tool (SAVES v6.0 package) (Figure 1C).

Using 3D structure imposition between the folded wildtype and mutant A1AT proteins, the C-atom structural coordinates were estimated in the form of RMSD scores by rotating them in three-dimensional space. No significant structural differences between the wildtype and all six mutant models of A1AT protein was observed at whole protein structure level as their RMSD scores fell less than 0.2 Å (Table 3). The RMSD values for F76S, S77F, L278P, E288V, G216C, and H358R are 0.038 Å, 0.028 Å, 0.029 Å, 0.039 Å, 0.049 Å, and 0.069 Å, respectively. Overall, the superimposition analysis of A1AT *protein* demonstrated that all six missense variants cause subtle structural changes in A1AT at the whole protein level (Figure 2). However, all mutant models show >2Å structural deviations at the residue level.

**TABLE 3:** 3D structural deviation of mutated *A1AT protein* structures *versus* wildtype represented in the form of RMSD values.

#	dbSNP ID	Amino acid variant	RMSD values (Å)	
			Whole structure	Amino acid
1	rs1555369172	F76S	0.038	2.4
2	rs55819880	S77F	0.028	2.9
3	rs756773408	G216C	0.029	3.1
4	rs1566753480	L278P	0.039	2.5
5	rs17580	E288V	0.049	2.8
6	rs1555367891	H358R	0.069	2.9

**FIGURE 2:**
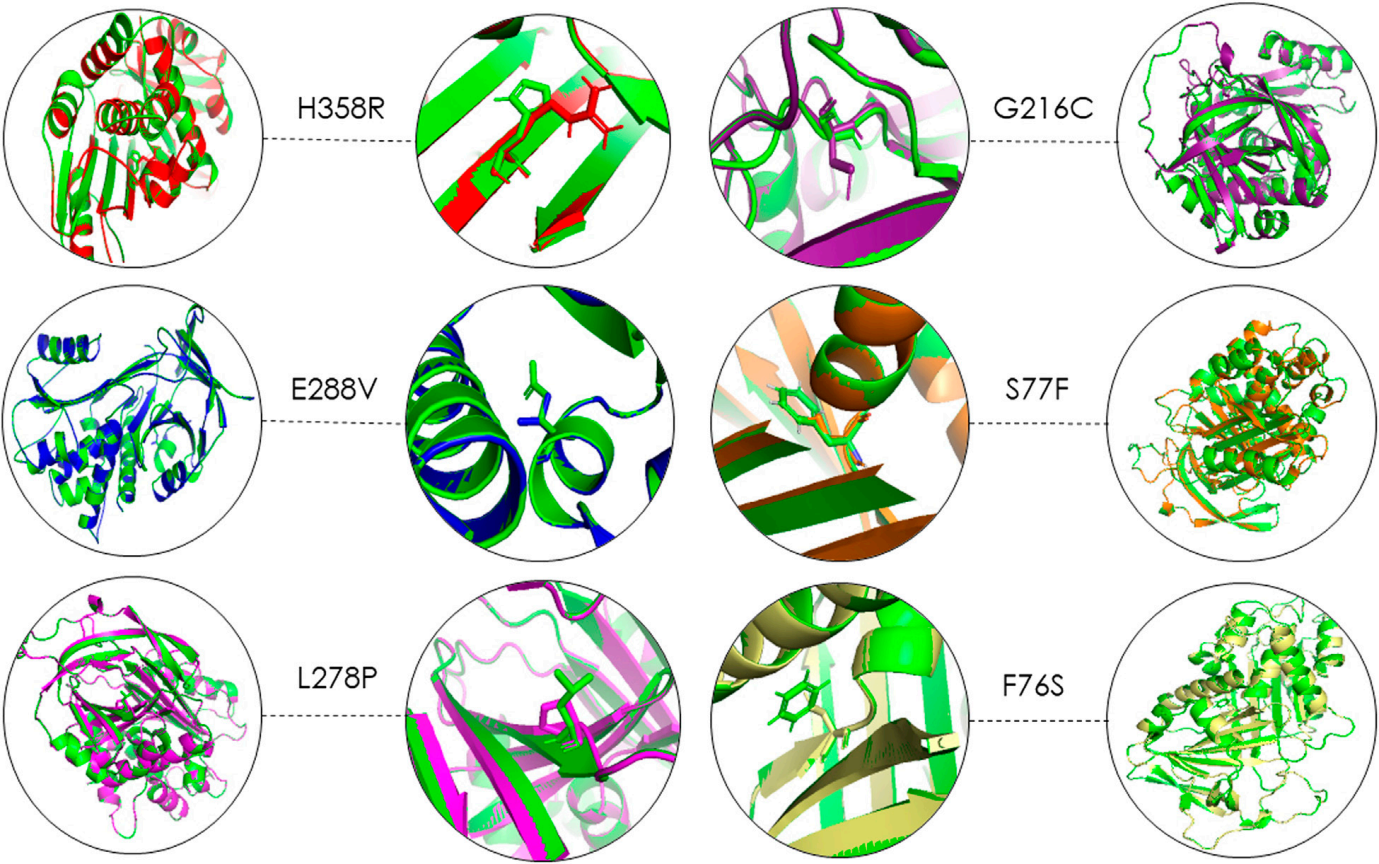
3D structure superimposition shows subtle variation in amino acid structures for all A1AT variants (Green = Wildtype).

### A1AT stability and secondary structure analysis

The function of the disease candidate protein will be affected by missense variants that negatively affect thermodynamic stability. Stability changes of A1AT mutated structures were analyzed by different tools to measure energy changes. The mCSM predicted F76S (−2.9 kcal/mol), S77F (−0.584 kcal/mol), L278P (−1.571 kcal/mol), G216C (−0.415 kcal/mol), and H358R (−1.74 kcal/mol) variants (5/6; 83.3%) are destabilizing the protein due to their negative free energy (ΔΔG) values. SDM also predicted F76S (−3.57 kcal/mol), G216C (−1.12 kcal/mol), L278P (−2.58 kcal/mol), and H358R (-1.69 kcal/mol) variants as destabilizing owing to their free energy values.

The DUET tool combines the output of both the mCSM and SDM tools to generate a consensual prediction. DUET webserver supported that 5/6 variants (83.3%) are destabilizing the A1AT structure because of their negative free energy values, i.e., F76S (-3.199 kcal/mol), S77F (−0.419 kcal/mol), G216C (−0.434 kcal/mol), L278P (−1.93 kcal/mol), and H358R (−1.842 kcal/mol). The destabilization data predicted that most of these variants are pathogenic as they disrupt the folding of the A1AT protein. Because of positive free energy values, E288V is, predicted by all the three tools (mCSM = 0.909 kcal/mol, SDM = 0.55 kcal/mol, and DUET = 1.333 kcal/mol), to further stabilize the A1AT *protein* structure (Table 4).

**TABLE 4:** Thermodynamic stability analysis of SERPINA1 missense variants.

#	dbSNP ID	Nucleotide substitution	Exon	Amino acid variant	mCSM (ΔΔG)	SDM (ΔΔG)	DUET (ΔΔG)
1	rs1555369172	c.274T>C	2/5	F76S	−2.9 kcal/mol (Destabilizing)	−3.57 kcal/mol (Destabilizing)	−3.199 kcal/mol (Destabilizing)
2	rs55819880	c.277C>T	2/5	S77F	−0.584 kcal/mol (Destabilizing)	0.74 kcal/mol (Stabilizing)	−0.419 kcal/mol (Destabilizing)
3	rs756773408	c.693G>T	2/5	G216C	−0.415 kcal/mol (Destabilizing)	−1.12 kcal/mol (Destabilizing)	−0.434 kcal/mol (Destabilizing)
4	rs1566753480	c.880T>C	3/5	L278P	−1.571 kcal/mol (Destabilizing)	−2.58 kcal/mol (Destabilizing)	−1.93 kcal/mol (Destabilizing)
5	rs17580	c.910A>T	3/5	E288V	0.909 kcal/mol (Stabilizing)	0.55 kcal/mol (Stabilizing)	1.333 kcal/mol (Stabilizing)
6	rs1555367891	c.1120A>G	5/5	H358R	−1.74 kcal/mol (Destabilizing)	−1.69 kcal/mol (Destabilizing)	−1.842 kcal/mol (Destabilizing)

**FIGURE 3:**
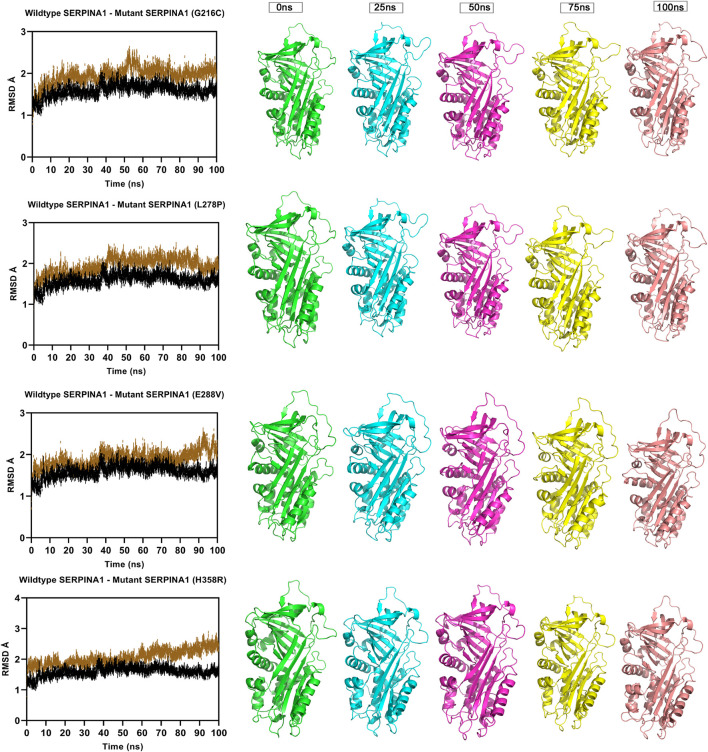
Molecular dynamics simulation measuring fluctuation in structural stability at the protein level for 4 A1AT variants (G216C, L278P, L288V, and H358R) when subjected to a force at different time intervals (measured at 0, 25, 50, 75, and 100 ns. ns = nanoseconds).

The secondary structure analysis provides information on how the mutant amino acid residue affects the substructures (alpha helices, loops, and beta pleated sheets) in the protein. The output of the secondary structure analysis predicted 2/6 (33%) of variants show alterations in the secondary structure of A1AT This includes L278P variant, which shortens the beta pleated sheet (Figure 3) and the S77F variant that increases the protein length as this variant is located at the junction with a loop region (Figure 3).

### MD simulation analysis

**FIGURE 4:**
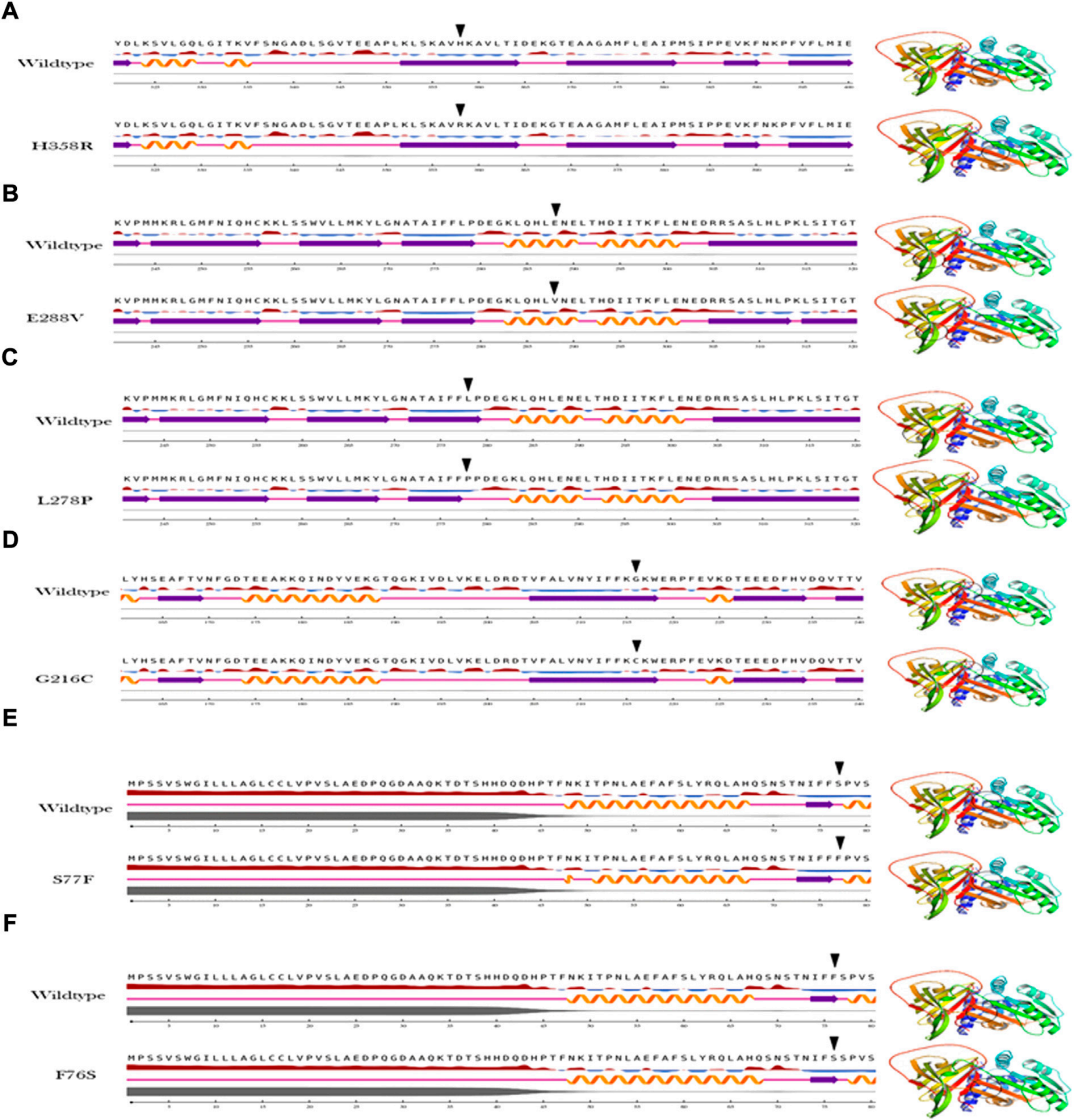
Secondary structure analysis output of the A1AT missense variants **(A–F)** in comparison with wildtype structure.

The Root Mean Square Deviation (RMSD), Root Mean Square Fluctuations (RMSF), and Secondary Structure Elements (SSE) were analyzed in all the six alpha-1 anti-trypsin models at 100 ns. Figure 4 represents the C-alpha RMSDs of wildtype and mutant alpha-1 anti-trypsin proteins for 100 ns. The RMSD analysis shows that four mutant structures (G216C, L278C, E288V, and H358R) significantly fluctuated compared to the wild-type alpha-1 anti-trypsin model. The wildtype model RMSD started at 1.4 Å at 0 ns and reached equilibrium at 50 ns, maintaining the RMSD range between 1.5 Å and 1.8 Å. Whereas the RMSD of the four mutant models increases steadily up to 10 ns, after 50 ns, it abruptly decreases from 0.2 Å to 0.42 Å and then increases to 1.8 Å at 70 ns, before fluctuating at 1.6 Å at the end of the simulation. In the H358R mutant model, a clear deviation was observed in the domain region, whereas in the other models, the helix 12 region showed more deviation compared to other secondary structural elements in the protein models. The mutations G216C, L278C, E288V, and H358R are localized near the RCL region of A1AT. After 50 ns, the β -sheets were opened (at RCL), allowing an increase in the stability of the motifs in the protein. We also discovered that the RCL region is stressed to a relaxing state during the simulation period.

**FIGURE 5:**
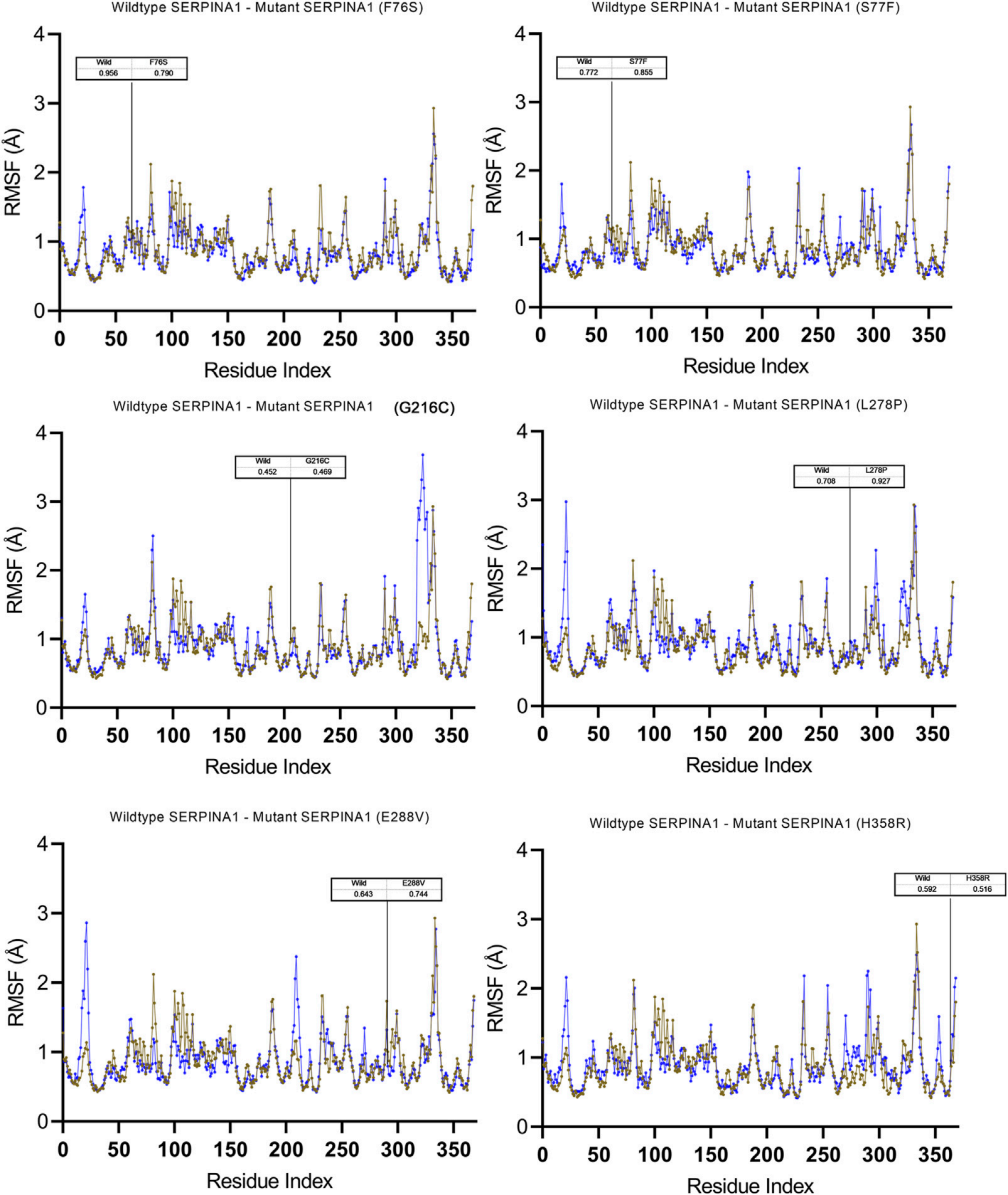
Molecular dynamics simulation measuring fluctuation in structural stability at the amino acid level for 6 A1AT variants (F76S, S77F, G216C, L278P, E288V, and H358R) after being subjected to a force.

To understand the flexible nature of mutated proteins, we performed RMSF analysis at 100 ns. The RMSF value over the entire simulation of six mutant models (F76S, S77F, G216C, L28P, E288V, and H358R) showed significant fluctuations. When compared to the wildtype model, the mutant models S77F, G216C, L278P, and 288V exhibit more fluctuations (>0.2 Å), whereas the mutant models of F76S and H358R exhibit more rigidity (>0.1 Å). The higher RMSF values of mutated models of S77F, G216C, L28P, and E288V support the calculated RMSD values. The secondary structure analysis was performed on mutated models of the alpha-1 anti-trypsin protein. Of the 6 mutated models, two models show a significant alteration in secondary structural elements. The mutated models S77F and G216C show >1% alterations in the secondary structural elements, whereas the remaining four models show lesser alterations in their β-strands (Figure 5).

### Molecular docking of A1AT-NE complex

**FIGURE 6:**
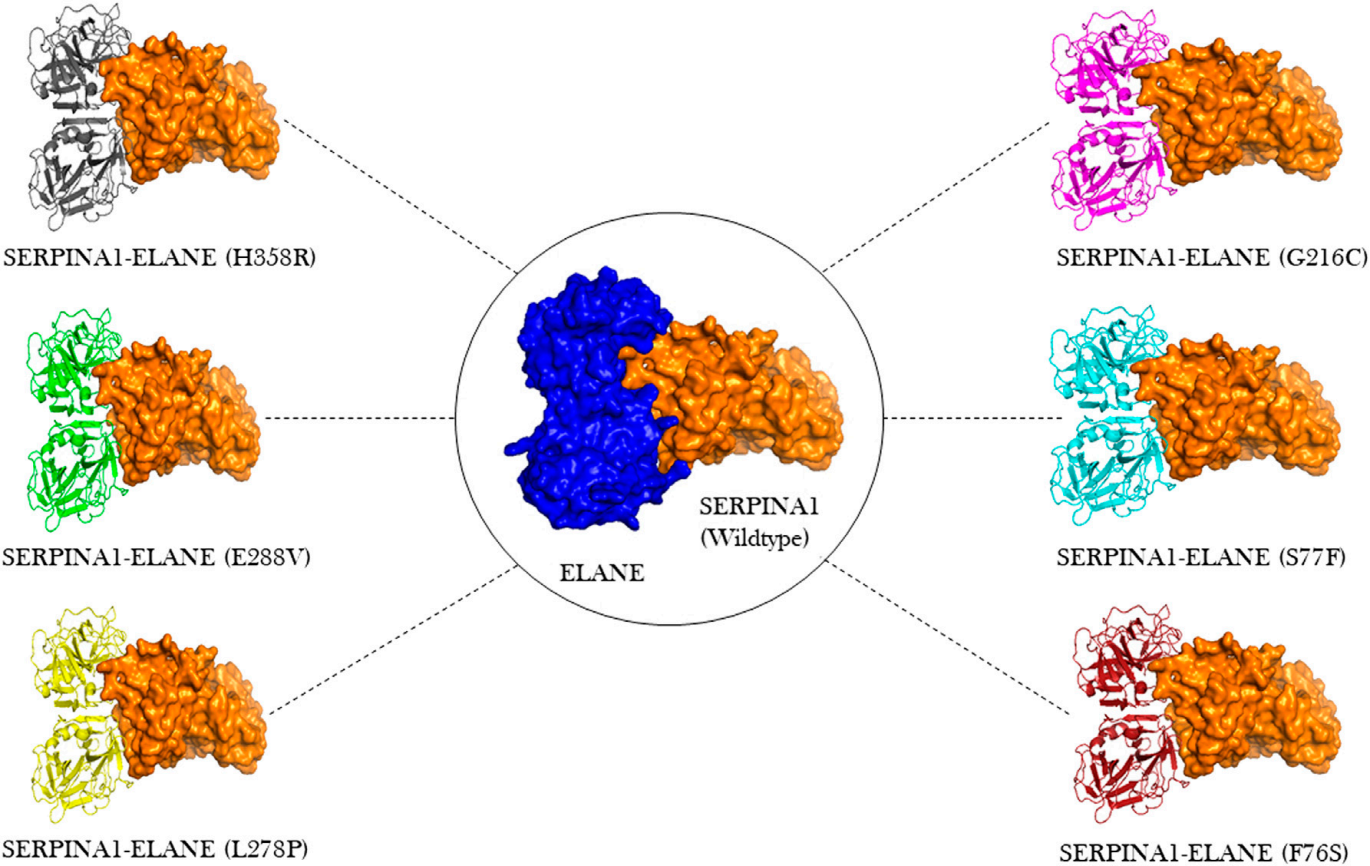
Molecular docking visualization output of the 6 A1AT variants and wild type protein showing their binding configurations with *NE* (neutrophil elastase).

ClusPro docking generated the best docking complexes of A1AT (receptor) and NE (ligand) with similar polarity and orientation based on their high-resolution models fitted to the electron microscopy density volumes above. The ClusPro software calculated the best docking pose based on highly populated clusters of low-energy models. Further, the best fit pose for each receptor-ligand complex was identified by the PIPER algorithm based on electrostatic and van der Waals scores (Figure 6). A1AT-NE mutated complexes have shown significant alterations in lowest central energy score (>-9.1 kcal/mol), compared to the wildtype-NE complex (Table 5). Therefore, major differences in binding configuration for all the six variants are predicted by this analysis. In the wildtype *A1AT protein* complex, the protein-protein interaction had the lowest free energy score of −914.9 kcal/mol, whereas the lowest free energy between F76S-NE complex is −941.2 kcal/mol, in S77F-NE is −944.6 kcal/mol, in G216C*-*NE is −941.5 kcal/mol, in L278P*-*NE is −945.4 kcal/mol, in E288V-NE is −941.1 kcal/mol, and in H358R-NE is −931.6 kcal/mol. The A1AT *protein* normally functions as a molecular mouse trap by having high affinity to its substrate and eliminates its target molecule in a suicidal fashion (Berclaz and Trapnell, 2006). Under A1AT mutant conditions, differences in binding affinities with NE were evident. The NE interacts at RCL loop in both wild and mutant state. However, the mutation at AA348 created a expansion of B-sheet and allowed perturbation of the Helix in the A1AT structure. This will allow the formation of protein-inhibitor covalent liked complex. Other mutations are not having any direct effect on the NE- A1AT inhibition. As a result of these findings, it is assumed that all six mutant models will tightly bind to their substrate, altering the way the alpha-1 antitrypsin protein functions inside the cell. The A1AT and protease molecular complex are recognized by hepatic receptors, which rapidly clear it from the blood circulation. A1AT has been shown to have a variety of different immunomodulatory actions in addition to its function as the main protease inhibitor, including an anti-inflammatory effect and the modulation of T- and B-lymphocyte functions.

**TABLE 5:** Cluspro predictions of A1AT-NE molecular complex binding energy scores.

#	Receptor-ligand	Cluspro weighted scores	
		Center score (K.cal/mol)	Lowest energy (K.cal/mol)
1	A1AT-NE (Wildtype)	−798.7	−914.9
2	A1AT -NE (F76S)	−793.0	−941.2
3	A1AT -NE (S77F)	−798.5	−944.6
4	A1AT -NE (G216C)	−798.1	−941.5
5	A1AT -NE (L278P)	−794.8	−945.4
6	A1AT-NE (E288V)	−792.4	−941.1
7	A1AT-NE (H358R)	−775.0	−931.6

## Discussion

A1ATD manifests clinically with emphysema in the lungs around the fourth to fifth decade of life, with a proportion of patients developing liver cirrhosis later due misfolded A1AT protein aggregates accumulating in hepatocytes (Kelly et al., 2010). The *SERPINA1* gene is located on chromosome 14q32.1 and has three untranslated exons (IA, IB and IC) and four coding exons (II–V). The first three exons regulate gene expression through three alternative transcription initiation sites: exons IA or IB in macrophages, exon IC in hepatocytes (Duvoix et al., 2014; Greulich et al., 2017; Tejwani and Stoller, 2021). Pathogenic variants in the *SERPINA1* gene underlie alpha-1-antitrypsin deficiency (A1ATD), which causes reduced protein levels. Many pathogenic *SERPINA1* variants associated with A1ATD have been reported in medical literature (Foil, 2021). Biological characterization of each genetic variant is impractical owing to their high number and time-consuming laboratory methods. In recent years, different researchers have shown the successful application of different computational methods like SIFT, Polyphen-2, M-CAP, and FATHHM in screening clinically pathogenic variants (Kelly et al., 2010; Hunt and Tuder, 2012; Lage, 2014). Computational biology-based pathogenicity prediction methods employ different support vector machine-based algorithms to identify deleterious variants from non-deleterious ones (Berclaz and Trapnell, 2006).

In the current study, computational predictions of SIFT, Polyphen, M-CAP, FATHMM, CADD and REVEL methods have confirmed all the six variants as pathogenic. However, computational predictions are often variable, based on the fact that each tool is trained on a unique variant data set (Thirumal Kumar et al., 2022; Thirumal Kumar et al., 2021; Abdel-Motal et al., 2018; Agrahari et al., 2019; Selvakumar et al., 2019).

This study found that two of the selected variants (F76S and S77F) fell in close range to the previously reported Trento variant of A1AT (E75V) (Miranda et al., 2017). The patient had a severe case of A1ATD with pathogenic intracellular polymer formation. The E75V variant affects the hydrogen bonding of the glutamic acid sidechain to the backbone of helix I which causes destabilization to the post helix I loop. This geometry has been shown to play a conserved role in preventing polymerization (Lomas et al., 1992). As a result of the expected geometry disturbance, F76S and S77F are expected to produce a polymerization phenotype similar to the Trento variant in affected individuals.

The evolutionary conservation analysis has identified that the H358R variant is in the Serpin conserved domain (344aa–369aa). This variant is reported in ClinVar as likely pathogenic. It is assumed that this variant might introduce a functional change in the protein conserved domain, but the secondary structure analysis for this variant did not show any clear alterations in secondary structural elements like α helices and β pleated sheets. Recent research has shown that missense variants *TYK2* (Pro1104Ala), *IL6R* (Asp358Ala), and *PTPN22* (Trp620Arg) are unaffected by secondary structural element (SSE) changes in their respective proteins. The other variants were reported on ClinVar as follows: F76S as pathogenic, S77F as pathogenic, G216C as likely pathogenic, L278P as pathogenic, and E288V as pathogenic. The pathogenicity prediction tools provide a qualitative support for damaging or not damaging effect but do not show additional details on the structural changes caused by the variants. *SERPINA1* missense alleles are associated with a significant reduction in A1AT serum levels because of the incorrect folding of the protein, poor stability, or degradation.

A1AT is a 52-kDa plasma glycoprotein with 394 amino acids. Its expression inversely correlates with expression of its binding partner NE [28]. The damaging effects of missense variants are better understood when their impact is studied at 3D structure level [29]. The superimposition of the investigated 3D protein structures onto the folded wildtype model is demonstrated to be a useful method for estimating root-mean-square deviation (RMSD), the average distance between backbone atoms of the superimposed variant and native protein structures [29]. In this investigation, we did not find major structural differences in the whole structure of A1AT in mutated state. However, all 6 missense variants cause subtle structural changes at the residue level. Amino acid substitutions often result in quantitative structural alterations that are accompanied by changes in fundamental physicochemical characteristics such as size, charge, side chains, molecular weight, and hydrophobicity. All of these modifications could affect the amino acid residues’ chemical bonding properties (hydrogen bonds, ionic bonds, and Vander wall interactions), which are necessary for keeping the A1AT protein molecule in its secondary (alpha helices, beta sheets, and coils), tertiary (3-dimensional form), and quaternary (protein subunit arrangement) structural conformations.

Thermodynamic stability can provide information about the nature of the A1AT’s altered function. The greater the negative free energy, the more destabilizing the variant. In this combination, the DUET algorithm predicted two variants with highest negative free energy for H358R (−1.842 K. Cal/Mol) and F76S (−3.199 K. Cal/Mol) to be as destabilizing. The H358R falls into the Serpin conserved domain and the F76S has changed from a large hydrophobic residue (phenylalanine) to a small hydrophilic residue (serine). This might push the protein region towards the external environment, altering the overall thermodynamic and structural orientation.

To get a deeper insight into how variants influence the stability of the A1AT protein structure on the molecular level, molecular dynamics simulations were performed for the wild type and mutant models. The output trajectory of the simulation at multiple intervals (0, 25, 50, 75, and 100 ns) was subjected to two analyses: RMSD and RMSF. The RMSF value over the whole simulation (100 ns) of six mutant models (F76S, S77F, G216C, L28P, E288V, and H358R) showed significant fluctuations. However, when subjected to a force, they showed a significant structural behavior change. The wildtype model had more fluctuations (>0.2 Å) than the mutant models S77F, G216C, L278P, and E288V, whereas the F76S and H358R variants were more rigid in nature (>0.1 Å). This finding demonstrates that missense variants could somehow introduce mild structural changes that could affect the behavior of the protein in its environment. The increase in RMSF values of mutated models of S77F, G216C, L28P, and E288V variants validated the calculated RMSD values. This suggests that these four variants must have some structural changes affecting the residue flexibility and behavior. Of the six mutated models, two models (S77F and G216C) showed a significant alteration in secondary structural elements. The S77F was already predicted in the previous secondary structure analysis as having an increased beta pleated sheet that is increased in length, giving more support to this finding. The secondary structure change can be attributed to the subtle changes giving rise to behavioral changes seen in MD simulations. However, we cannot assume it is the exact cause of this altered behavior.

Protein-protein interaction analysis is an important approach in understanding the variant impact on structural features of disease candidate proteins of genetic diseases (Lage, 2014). The reduction or increase in the binding affinity of a protein induced by residue alterations can make proteins lose their function and cause disease. The A1AT protein is the primary protease inhibitor in the human body, acting on a variety of targets including trypsin, collagenase, macrophage cathepsin, tissue kallikrein, factor IX and other molecules (Berclaz and Trapnell, 2006). However its main function is to keep the neutrophil elastase in balance during in inflammation or infection through its inhibitory action (Hunt and Tuder, 2012). In this analysis, molecular docking has yielded a similar range of lowest energy (−931.6 K. Cal/Mol to−944.6 K. Cal/Mol), which predicts a significant difference versus the wildtype protein (−914.9 K. Cal/Mol). This results in a strong binding to *NE* (−16.7 K. Cal/Mol to 30.5 K. Cal/Mol deviation range), most likely causing a functional alteration. The binding configuration was chosen based on the best score, which could not have been compared to an available crystal structure. So, the actual amount was only an estimation.

## Conclusion

This study concludes that computational methods like SIFT, PolyPhen, FATHMM, M-CAP and REVEL tools are very helpful in prioritizing *SERPINA1* loss-of-function pathogenic variants. These tools have lot of promise in screening Alpha-1-antitrypsin deficiency causative variants from next-generation sequencing data. It is important to note that laboratory experimental methods are required for definitive answers to the questions asked in this analysis. This present analysis highlighted general structural abnormalities caused by the reported missense variants of *SERPINA1*. The structural and stability prediction methods used in this study have shown how loss-of-function pathogenic variants could induce structural drifts, free energy value fluctuations, and alter the conformational dynamics of the *A1AT* protein molecule. The SERPINA1 mutations result in an unstable intermediate structure that is responsible for the β sheet-A opening, which can accept the RCL of another A1AT molecule to form a loop-sheet dimer. The latter can then be extended to form longer chains of loop-sheet polymers. These models are based on the “classic” loop-sheet model in which serpin polymers are formed by the intermolecular linkage of the reactive loop of one molecule with the β-sheet A of another. The findings from molecular docking have demonstrated how most missense variants negatively impact the affinity of NE and A1AT binding in a molecular complex, lowering A1AT functionality and contributing to its deficiency. Taken together, our computational approach offers an extra layer to study the deleterious potential of *SERPINA1* genetic variants from the structure and function context. Our findings recommend implementing computational variant assessment as a pre-invitro phase in improving the genomic medicine for A1ATD patients carrying *SERPINA1* pathogenic variants.

## Data Availability

The datasets presented in this study can be found in online repositories. The names of the repository/repositories and accession number(s) can be found in the article/Supplementary Material.
