# Supplementary figures and images for: Structural characterization and conformational dynamics of alpha-1 antitrypsin pathogenic variants causing alpha-1-antitrypsin deficiency
Source: Front Mol Biosci. 2022 Nov 24;9:1051511. doi: 10.3389/fmolb.2022.1051511 (PMC9730039; doi:10.3389/fmolb.2022.1051511)

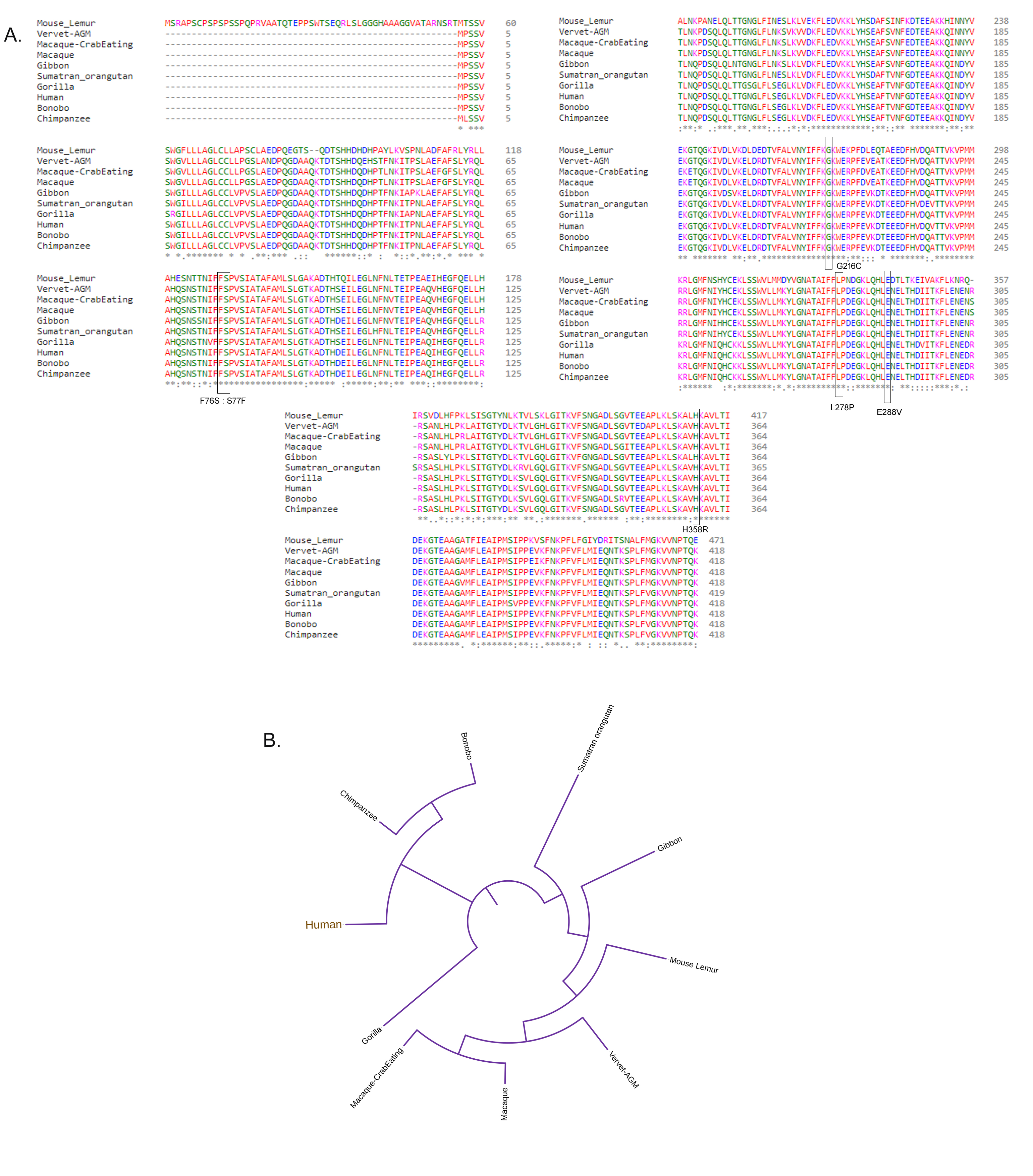

Supplement: Supplementary file 1 [file Image1.JPEG]
